# Scientific

**Published:** 1889-01

**Authors:** 


### SCIENTIFIC ITEMS.

**Curious Chinese Notions.**—Both savage and semi-barbarous people have always exhibited a great repugnance to any surgical operation, however necessary, which involves amputation. The *North China Herald*, in commenting upon this circumstance, points out that the Chinese have always shown this repugnance, not on account of fear or pain, for they are patient under all kinds of physical suffering, but because they loook upon it as a duty to keep the body intact. If they submit to the amputation of a limb, they invariably ask for the severed member, and keep it in a box, to be buried in due time with the owner. Sometimes they will actually eat it, thinking it only right that that which has been taken from the body should be returned to it. On the same principle, an extracted troth will be carefully preserved, or ground to powder and swallowed in water. Another curious phase of the same idea is seen in the belief that a sick parent can be cured by broth made from flesh cut from a living child, and it is looked upon as a sign of filial piety for the child to submit himself to an operation for that purpose. The child is supposed to be of the vital essence of the parent, and if a portion of this essence is returned to the fountain-head, the parent will be greatly strengthened. The peace-loving nature of the Chinese is said to be largely due to this respect for the human body.—*Chamber's Journal.*

**The Nearest Star.**—The distances of the stars are ascertained in the same manner as those of the sun and planets; that is by parallax. Instead, however, of taking two stations at different parts of the earth's surface, and laying down a base line between them, we take the diameter of the earth's orbit, or 183,000,000 miles, as the base; the observations being taken at intervals of six months. Even with this immense line, however, the parallax is so small that it can only be detected by the most careful observations and accurate instruments. The parallax of about a dozen stars has now been ascertained, and is found to vary between 0.919 sec. and 0.046 sec. The star *a* Centauri is the nearest to the earth, and its distance is estimated at 20,496,000,000 miles; while the average distance of the first magnitude is probably three times as great as this.

**Electric Prostration.**—Several cases of this new malady are reported from Creusot, France. It affect workers under electric lights. The light exceeds 100,000 candle power, and it appears that it is this excess of light, and not the heat, which produces the nervous symptoms. A painful sensation in the throat, face, and temples is first noticed, then the skin becomes coppery red, and irritation is felt about the eyes, much lachrymation ensues, and the symptoms then disappear, while the skin peels off in five days. The effects are comparable to those produced by walking over fresh snow in the sunlight, and may be regarded as a sort of "sun-burning."—*Lancet.*

**Lightning by means of Accumulators.**—At Springfield, Mass., the electric light company have recently put into their works on Taylor Street the system of the Electrical Accumulator Company, of New York, composed of 378 large cells, which take up a floor space of about 20 by 13 feet, and they stand about 8 feet high. The company are able to store electricity enough in the accumulators to run 500 lights ten hours. In this way they yre able to do more work with the same amount of engine power, as the engines can be used to store up electricity during the day for use in the night, and then the same motive power can be used to propel the arc dynamos at night.

**A Large Organ.**—A correspondent of *La Science en Famille* states that in the Protestant church at Libau (Russia) there is an organ which occupies the whole width of the church, about sixty feet, and which has 131 registers,, 8,000 pipes, and 14 bellows of large size. It has 4 harpsichords and one pedal. The largest pipe is formed of planks 3 inches thick and 31 feet in length, and has a section of 7 square inches, and weighs 1,540 pounds. Besides the 131 registers, there are 21 accessory stops that permit of combining various parts of the instrument without having direct recourse to the register. By a special pneumatic combination, the organist can couple the four harpsichords and obtain surprising results. For the sake of comparison, the following large instruments of this kind may be cited: Organ of the Cathedral of Riga, 125 registers; Garden City Cathedral 120; St. Albert Hall, London, 100; Cathedral of Ulm 100; St. George's Hall, Liverpool, 100; Notre Dame, Paris, 90; Boston Cathedral 86; Cathedral of Schwerin 85; St. Nicholas Church, Leipzig, 85; Cologne Cathedral 42.—*Scientific American.*

**Eels that Scale Precipices.**—One of the most novel sights in the spring of the year, at the rocks of the Willamette Falls, is the swarms of gyrating eels. They are friskiness itself, and show a low order of intelligence. If you put your hand in the water over the eels, or spit in it, instantly they are gone. But poke a stick down among the snaky things, and they do not notice it. The sense of smell seems to be their main guard against danger. Like Salmon, they do their level best to dart up the rocks in order to ascend the river, and with good success. Says a fisherman:

"I have seen as many as a hundred bushels of eels hanging on the rocks at one time by the suckers of the mouth. They would wiggle and flutter their tails, and by the momentum thus obtained, letting go with their suckers, jump up about six inches higher. I caught about forty barrels last season that I salted and sold to the Columbia fishermen for bait. I picked them off the rocks with a fish hook tied to a pole. I started at the bottom row of hanging eels, and would silently pick off barrel after barrel. The upper rows hadn't sense enough to perceive the enemy. I have caught eels in the headwaters of the Santiam, in the Cascade Mountains. Suppose they had swum up from the Willamette."—*Oregon City Courier.*

